# Low BMD is uncommon in pediatric mastocytosis: a DEXA- based cohort study

**DOI:** 10.1007/s00431-026-06953-z

**Published:** 2026-04-29

**Authors:** Antje Geypen, Chantal Jhinkoe, Suzanne G. M. A. Pasmans, Nicolette J. T. Arends

**Affiliations:** 1 Department of Dermatology, Erasmus MC, Rotterdam, The Netherlands; 2 Pediatric Allergology, Erasmus MC, Rotterdam, The Netherlands; 3 Erasmus University, Rotterdam, The Netherlands

**Keywords:** Pediatric mastocytosis, Bone mineral density, Cutaneous mastocytosis

## Abstract

Mastocytosis is a disorder characterized by abnormal mast cell proliferation, leading to cutaneous mastocytosis (CM) or systemic mastocytosis (SM) when internal organs are affected. While adult mastocytosis is associated with osteoporosis, data on bone mineral density (BMD) in children are lacking. To our knowledge, this is one of the first studies investigating BMD in pediatric mastocytosis. We conducted a prospective cross-sectional study including clinical and laboratory data from a database (2004-2025), covering mastocystosis subtype, serum tryptase, and c-kit mutation status. BMD was measured in children aged 4-18 years using dual-energy X-ray absorptiometry (DEXA) at the lumbar spine and total body. 76 children were included (73 CM, 3 SM [ISM]). Low BMD (Z-score ≤ –2) was observed in 6.8% of CM patients, significantly more prevalent than in the general pediatric population (*p* = 0.0014). However, all normalized spontaneously during follow-up. None had osteoporosis. ISM patients had significantly higher tryptase levels but normal BMD. BMD was not correlated with disease specific characteristics, bone pain or fractures.

*Conclusion*: Our findings suggest that bone involvement is not a major concern in pediatric mastocytosis. DEXA scans could potentially be reserved for children with suspected systemic involvement. Future longitudinal studies are necessary to further elucidate bone involvement in these patients.

**Table:** 

**What is Known:** • *Mastocytosis is a rare disorder, associated with osteoporosis and fractures in adults. Data on bone density in the pediatric population are lacking.*	
**What is New:** • *Our results suggest that bone involvement is not a major concern in pediatric mastocytosis.*

**What is Known:**

• *Mastocytosis is a rare disorder, associated with osteoporosis and fractures in adults. Data on bone density in the pediatric population are lacking.*

**What is New:**

• *Our results suggest that bone involvement is not a major concern in pediatric mastocytosis.*

## Introduction

Mastocytosis is a rare disorder characterized by abnormal proliferation and activation of atypical mast cells, which can accumulate in various tissues such as the skin, bone marrow, liver, spleen, lymph nodes, and gastrointestinal tract. [1] When mast cells accumulate only in the skin, it is classified as cutaneous mastocytosis (CM), while internal organ involvement results in systemic mastocytosis (SM) [1–3]. The estimated prevalence of mastocytosis is about 1 per 10,000 persons in the USA [2]. Approximately twothirds of diagnoses are made in childhood. Eighty percent of children present with limited cutaneous disease (CM) [1, 2, 4–6]. Most children present with CM at a young age. Skin lesions typically regress spontaneously around puberty, but progression to SM does rarely occur, particularly in children with late-onset disease. SM without skin involvement has not been reported in children [1, 2, 7, 8].

Activating *KIT* gene mutations play a central role in the pathogenesis of mastocytosis. More than 80% of adult patients carry the *KIT* D816V mutation in exon 17, while childhood-onset mastocytosis knows a wider variety of *KIT* mutations; only 35% express *KIT* D816V, while 40% express other *KIT* mutations affecting exon 8, 9, or 11 and 25% have no detectable *KIT* mutations (*KIT* wild-type) [3, 9]. The presence of *KIT* D816V mutation is associated with an increased risk of ISM [1, 9].

**Fig. 1:**
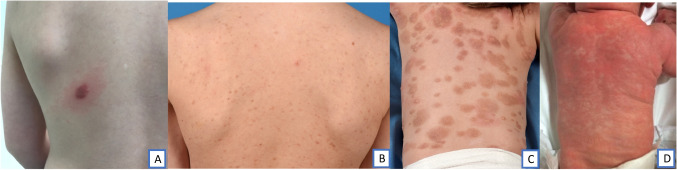
Clinical pictures of different CM subtypes [12]. A mastocytoma and positive Darier’s sign, B monomorphic MPCM, C polymorphic MPCM, D DCM. These pictures are published with explicit permission from the parents/guardians.

CM includes three forms (figure 1), with maculopapular cutaneous mastocytosis (MPCM) being the most common. Monomorphic MPCM (mMPCM) presents as multiple small lesions of similar size and shape, while polymorphic MPCM (pMPCM) knows irregular large lesions with variable size and shape. The polymorphic variant is predominant in children and is correlated with a favorable prognosis, while mMPCM seems to be correlated with higher tryptase levels and disease persistence into adulthood. [1, 2] Indeed, mMPCM and SM are the most common forms of adult mastocytosis [10, 11].

Symptoms arise from the release of mast cell mediators and range from asymptomatic to severe. They include itching, flushing, headaches, wheezing, gastrointestinal symptoms, bone pain, arthralgia, cognitive difficulties, and depression [1, 13]. The risk of anaphylaxis in children is < 1–10% [1, 13], which is higher compared to the general population (0.02–0.05%) [14]. Symptoms are more frequent in extensive skin involvement or systemic disease [1, 9, 10].

 The diagnosis is based on clinical examination and the presence of Darier’s sign. In order to define the CM subtype, detailed evaluation of skin lesion characteristics is necessary (Table 3) [1]. A thorough clinical examination with assessment of hepatosplenomegaly is needed. Additionally, the tryptase level and presence of *KIT* D816V mutation will be determined in peripheral blood. A skin biopsy (e.g., in atypical presentations or bullous disease), bone marrow biopsy, extensive sequencing of the whole c-kit gene, or an abdominal ultrasound might help in the diagnosis. Treatment is usually symptomatic, focusing on avoiding triggers and alleviating symptoms caused by mast cell mediator release, and long-term prognosis is favorable. Treatment includes antihistamines, topical or systemic corticosteroids, and/or leukotriene antagonists. Specific therapies are reserved for anaphylaxis management and systemic disease [1, 2, 9, 15–18]. All patients require regular follow-up due to the risk of systemic mastocytosis [1, 15].

Adult mastocytosis patients are also at risk for bone involvement (including osteopenia, osteoporosis, and osteosclerosis) due to chronic mast cell activation and release of mediators like histamine, tryptase, and cytokines, and to a lesser extent, to neoplastic infiltration of bone marrow [19]. Data on bone density in childhood mastocytosis is sparse [20, 21]. Pediatric osteoporosis is defined by (1) the combination of a bone mineral density (BMD) Z-score ≤ − 2 and a significant fracture history (either two or more long bone fractures before the age of 10 years, or three or more long bone fractures before 19 years); or (2) one or more vertebral compression fractures in the absence of high energy trauma or local disease irrespective of BMD. DEXA is the technique of choice for assessing BMD in children, including measurements of the lumbar spine (LS) and the total body (TB) [22].

To our knowledge, this is one of the first studies to investigate BMD in children with mastocytosis [20].

## Methods

This prospective cross-sectional pilot study was conducted at Erasmus MC - Sophia Children’s Hospital (Rotterdam), a national expert center for pediatric mastocytosis in the Netherlands. This study was performed in line with the principles of the Declaration of Helsinki. Ethical approval was obtained from our hospital ethics committee (METC-2025–0665) [23].

Using a longitudinal patient database (April 2004–May 2025), patients underwent standardized follow-up, including serum tryptase levels and c-kit D816V mutation testing (via ASO-qPCR and ddPCR, which have been reported to detect mutant alleles at levels of 0.003–0.02% and 0.03% variant allelic frequency (VAF), respectively [24, 25]) according to the Dutch guideline [26]. As part of our study protocol, DEXA scans were performed between 2017 and 2025 as part of standard follow-up in children aged 4–18 years. Selection of patients was guided by practical considerations, such as the child’s ability to undergo the procedure (e.g., the ability to remain still during the procedure) and alignment with the inclusion period. Clinical and demographic data, like the age at onset, number of skin lesions, comorbidities, number of reported symptoms (including itching, fatigue, dizziness, flushing, headache, runny nose, dyspnea, chest pain, palpitations, stomach ache, nausea and vomiting, diarrhea, reflux, and bone pain), and fracture history, were extracted from medical records. The mastocytosis subtype was defined according to clinical features (Tables 1 and 2).

**Table 1:** Classification of cutaneous mastocytosis [3]

Cutaneous mastocytosis (CM)
1. Maculopapular cutaneous mastocytosis (MPCM)
2. Diffuse cutaneous mastocytosis (DCM)
3. Solitary mastocytoma

**Table 2:** Features useful to identify subtypes and variants of CM [1]

Features		Subtype
**Number of lesions**	≤ 3	Mastocytoma
	> 4	MPCM
**Appearance of the lesions**	Small, round, mostly flat, brown or red maculopapular lesions	Monomorphic MPCM
	Larger, brown to red heterogeneous lesions of different sizes, with macular, papular, plaque-type or nodular aspects	Polymorphic MPCM
**Overall skin appearance**	Erythroderma and generalized pachyderma (thickened skin). Extensive spontaneous blistering with erosions. Extensive bullous lesions and small vesicles	DCM
**Localization of lesions**	Symmetrical distribution on the body, without involvement of the central face, palms, and soles of the feet	Monomorphic MPCM
	Asymmetric distribution with involvement of the head, particularly the lateral parts of the forehead, the neck and extremities	Polymorphic MPCM

We aimed to evaluate bone mineral density (BMD) in children aged 4–18 with various subtypes of mastocytosis and assess differences across subtypes. BMD was measured by dual-energy X-ray absorptiometry (DEXA) at the lumbar spine (LS) and total body (TB), expressed as age- and sex-adjusted *Z*-scores. Low BMD was defined as a *Z*-score ≤ − 2 SD in one or two measurements.

Statistical analyses were performed using IBM SPSS 29. Tables and graphs were made with the same software. Differences among mastocytosis subtypes were tested using Kruskal–Wallis and post hoc Mann–Whitney *U* tests (with Bonferroni correction). Spearman’s correlation was used to evaluate associations between variables, with a significance level set at *p* < 0.05.

## Results

### Patient characteristics

**Fig. 2:**
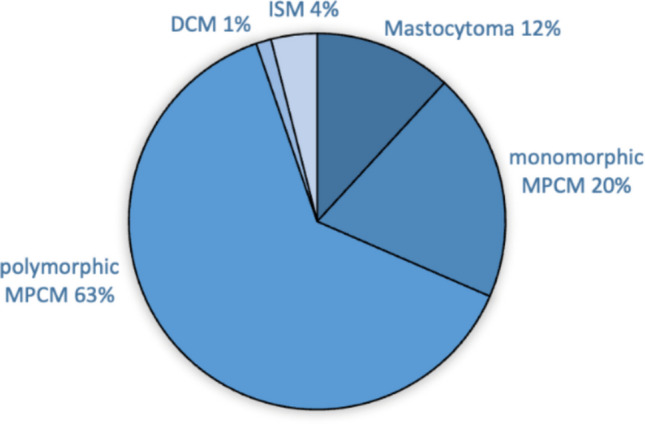
Types of mastocytosis in dataset

In 76 patients, of a total of 229 children with mastocytosis who were included in the database, a DEXA scan was performed. Among these, 73 patients were diagnosed with CM (96%) and 3 (4%) with SM (ISM). Statistical analysis was performed separately in both subsets of patients. The percentages of different subtypes in our database are depicted in Fig. 2.

The cohort of CM patients consisted of 45 males (61.6%) and 28 females (38.4%). Mastocytoma was present in 12.3% (*N* = 9) of cases. MPCM was the most common form (*N* = 63, 86.3%), with 20.5% (*N* = 15) of patients classified as monomorphic and 65.8% (*N* = 48) of patients classified as polymorphic. DCM was the least common, accounting for 1.4% (*N* = 1) of patients.

A positive Darier’s sign was observed in 88.7% (*N* = 63) of assessed patients. Diagnosis was confirmed by skin biopsy in 60.3% (*N* = 44) of patients. Peripheral blood *KIT* D816V was tested in 77.8% of mastocytoma patients (*N* = 9), 92.1% of MPCM patients (*N* = 63), and 100% of DCM and ISM cases (*N* = 3, *N* = 1, respectively). Peripheral blood *KIT* D816V was present in 26.1% of tested patients, with the highest frequency in mMPCM (60%). In mastocytoma, pMPCM, and DCM, 0%, 14%, and 0% tested positive, respectively. All three (100%) ISM patients tested positive for this mutation.

Baseline tryptase levels varied significantly per subtype (Kruskal–Wallis H, *χ*^2^(4) = 20.209, *p* < 0.001) with a median level of 6.1 ng/mL (4.8 ng/mL in mastocytoma patients, 10.1 ng/mL in mMPCM, and 5.9 ng/mL in pMPCM). Pairwise comparisons using Bonferroni correction revealed significantly higher baseline tryptase levels in mMPCM patients than in pMPCM (adjusted *p* = 0.038) and ISM patients had higher baseline tryptase levels than mastocytoma and pMPCM patients (adjusted *p* = 0.025 and *p* = 0.018 respectively). No other pairwise comparisons reached statistical significance. The ISM cohort consisted of three patients. The age of onset of cutaneous symptoms ranged from 1 to 48 months, while diagnosis of systemic involvement took place between the age of 2–17 years. The 3 ISM patients all had extensive skin involvement. Mean tryptase levels ranged from 42.6 to 85.9 ng/mL. All three patients had a peripheral blood *KIT* D816V mutation, and the presence of this mutation was confirmed in bone marrow samples. At the time of inclusion in the database, none of these patients exhibited B or C findings, as defined by the revised WHO criteria [27], and all were consequently classified as having ISM.

Anaphylaxis occurred in 3 (3.9%) patients, among which were 1 pMPCM and 2 mastocytoma patients. The anaphylaxis triggers were medication for the pMPCM patient and food or insect bites for the mastocytoma patients. Additional patient characteristics are depicted in Table 3.

**Table 3.** Clinical characteristics per subtype and DEXA scan results

	Mastocytoma, 9	mMPCM, 15	pMPCM, 48	DCM^1^, 1	ISM, 3	Total, 76
Sex, male	7 (77.8%)	8 (53.3%)	30 (62.5%)	0	2 (66.6%)	45 (61.6%)
Age of onset, months, median (range)*	3 (0–36)	8 (0–144)	4 (0–60)	1^1^	1 (1–48)	4 (0–144)
Median age at DEXA, years (range)	6 (5–13)	10 (4–18)	7 (4–16)	5 (-^1^)	8 (5–17)	7 (4–18)
**Normal BMD**	9 (100%)	14 (93.3%)	44 (91.7%)	1 (100%)	3 (100%)	68 (75.7%)
DEXA TB^2^, *median (IQR)*	− 0.4 (− 1.4 to 0.5) (*n* = 9)	0.2 (− 0.6 to 0.6) (*n* = 14)	− 0.05 (− 0.7 to 0.6) (*n* = 44)	1.2 (*n* = 1)	− 0.4 (*n* = 3)	− 0.1 (− 0.7 to 0.4) (*n* = 71)
DEXA LS^3^, *median (IQR)*	− 0.65 (− 1.1 to − 0.1) (*n* = 8)	− 0.25 (− 1.0 to 0.5) (*n* = 14)	− 0.2 (− 0.9 to 0.5) (*n* = 43)	− 0.6 (*n* = 1)	− 1.2 (*n* = 3)	− 0.2 (− 0.9 to 0.5) (*n* = 69)
Skin lesions, *N*						
0–10	9 (100%)	0	4 (8.33%)	0	0	13 (17.11%)
10–20	0	1 (6.67%)	4 (8.33%)	0	0	5 (6.58%)
20–30	0	4 (26.67%)	0	0	0	4 (5.26%)
30–40	0	0	2 (4.17%)	0	0	2 (2.63%)
40–50	0	0	1 (2.08%)	0	0	1 (1.32%)
> 50	0	8 (53.33%)	29 (60.42%)	1 (100%)	3 (100%)	41 (53.95%)
D816V positive in peripheral blood, *N* (%)	0	9 (60%)	6 (12.5%)	0	3 (100%)	18 (23.7%)
Median tryptase, ng/mL (IQR)	5.2 (2.41–7.99)	16.3 (9.94–22.77)	6.1 (3.78–8.38)	24.9 (-^1^)	61.5 (-^4^)	7.1 (4.90–12.08)
Bone pain, *N*	1 (11.1%)	0	7 (14.58%)	0	2 (66.67%)	10 (13.16%)
**Low BMD**	0	1 (6.7%)	4 (8.3%)	0	0	5 (6.8%)
DEXA TB^2^, *median (IQR)*	-	− 1.9 (*n* = 1)	− 2.3 (− 2.3 to − 2.15) (*n* = 4)	-	-	− 2.3 (− 2.3 to − 2.0) (*n* = 5)
DEXA LS^3^, *median (IQR)*	-	− 2.0 (*n* = 1)	− 0.65 (− 1.3 to 0.2) (*n* = 4)	-	-	− 1.1 (− 1.7 to 0.1) (*n* = 5)
Skin lesions, *N*						
0–10	-	0	1 (2.08%)	-	-	1 (1.31%)
30–40	-	0	1 (2.08%)	-	-	1 (1.31%)
40–50	-	0	1 (2.08%)	-	-	1 (1.31%)
> 50	-	1 (6.57%)	1 (2.08%)	-	-	2 (2.63%)
D816V positive in peripheral blood, *N*	-	0	0	-	-	-
Median tryptase, ng/mL (IQR)	-	-	5.83 (4.24–11.48)	-	-	-
Bone pain, *N* (%)	0	0	1 (2.08%)	0	0	1 (1.31%)

*IQR*, interquartile range

*Data available from 58 patients, percentages are calculated based on the number of patients with available data

^1^Only one DCM patient in analysis; results are based on this one patient

^2^* TB,* total body

^3^*LS,* Lumbar spine

^4^Calculation of IQR not possible due to low sample size

## DEXA scan results

### CM patients

**Fig. 3:**
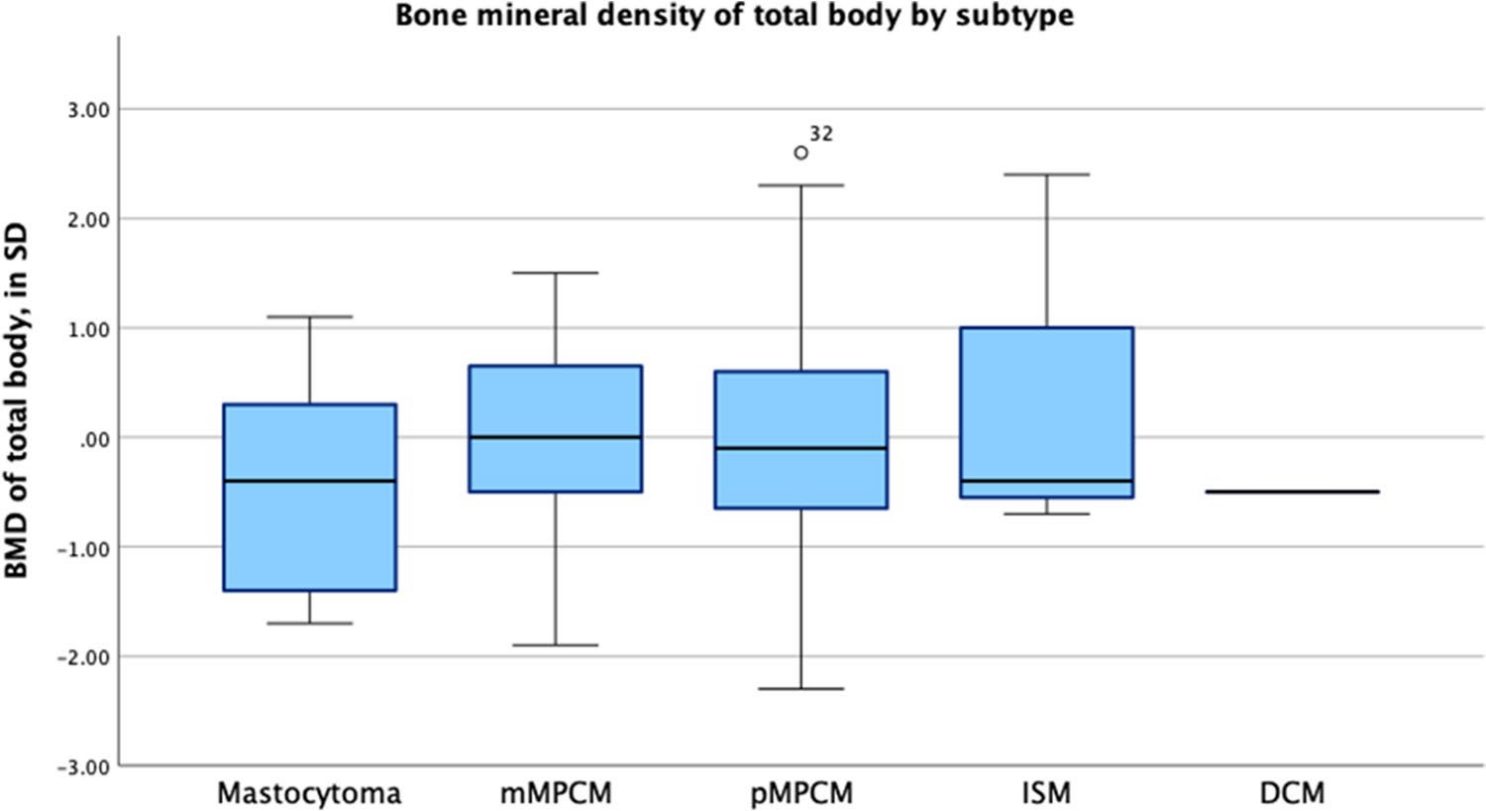
BMD *Z*-scores of the total body (TB)

**Fig. 4:**
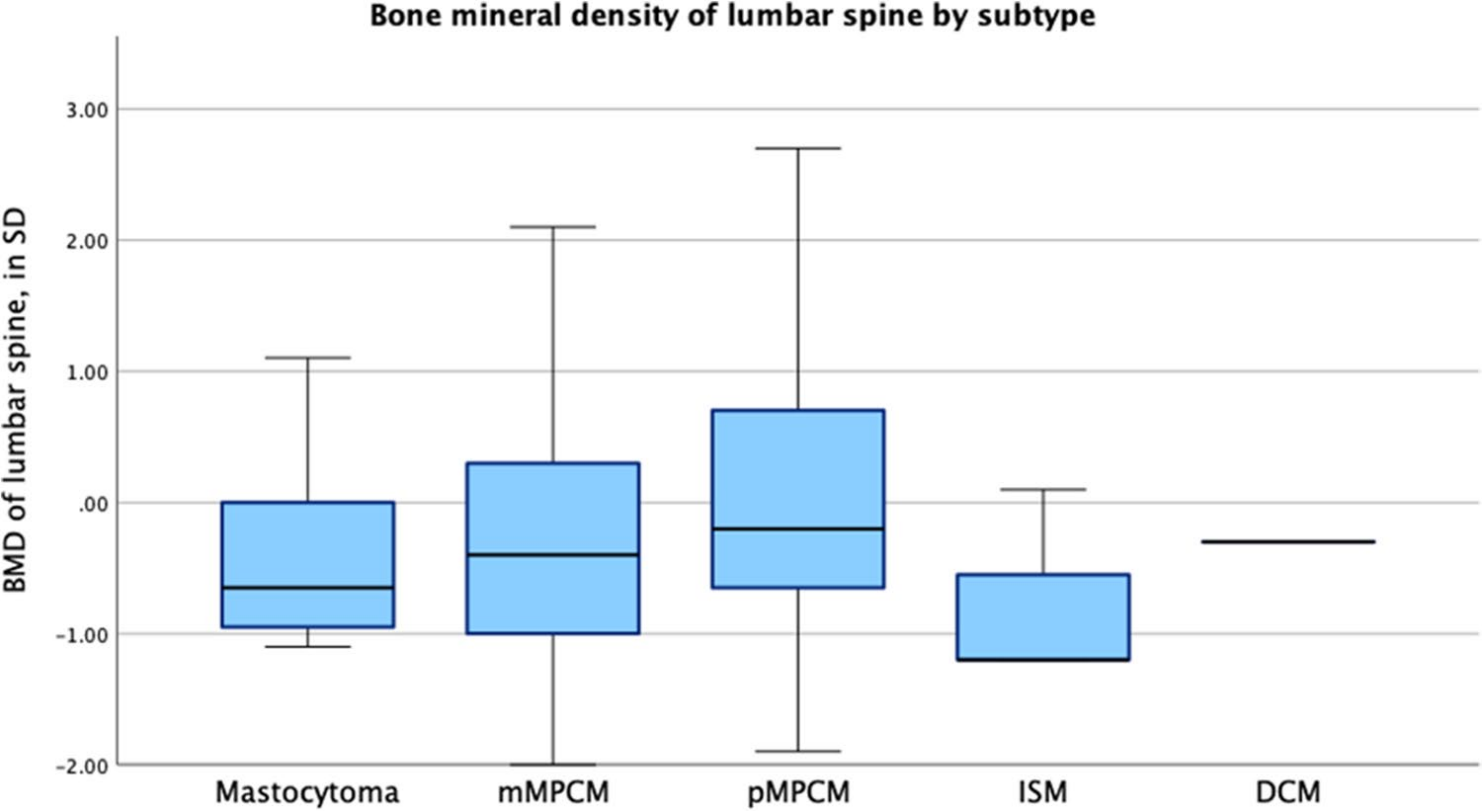
BMD *Z*-scores of the lumbar spine (LS)

Patient characteristics are demonstrated in Table 3. BMD *Z*-scores are illustrated in Figs. 3 and 4.

The median BMD *Z*-score of the TB was − 0.1 (IQR − 0.65 to 0.55), while the median BMD *Z*-score of the LS was − 0.1 (range − 0.9 to 0.5). BMD *Z*-scores of the TB and LS did not significantly differ across CM subtypes (BMD of TB in SD: *H* = 1.942, *p* = 0.584; BMD of the LS in SD: *H* = 2.158, *p* = 0.540).

Low BMD (*Z*-score ≤ − 2 in TB and/or LS, osteopenia) was identified in 6.8% (*N* = 5/73). This was significantly higher than the estimated prevalence of 2–3% in the general healthy pediatric population based on the standard distribution of *Z*-scores and pediatric DEXA reference data (*z* = 2.98, *p* = 0.0014) [22]. Notably, all five cases of low BMD occurred in patients with MPCM, and all tested negative for the *KIT* D816V mutation. Among the five patients with low BMD, one female patient with mMPCM underwent the scan at the age of 17. Four male patients with pMPCM had scans between the ages of 4 and 7 years. No significant correlations were observed between osteopenia and CM subtype, tryptase levels, *KIT* mutation, age of onset, or number of skin lesions.

Moreover, BMD did not differ significantly between patients with and without a peripheral blood *KIT* D816V mutation. The median TB BMD *Z*-score was − 0.1 (IQR − 0.9 to 0.50) in D816V-negative patients and − 0.3 (IQR − 0.50 to 1.20) in *KIT* D816V-positive patients (*U* = 406.5, *p* = 0.473). Similarly, the median LS BMD *Z*-score was − 0.2 (IQR − 0.90 to 0.50) in *KIT* D816V-negative patients and − 0.2 (IQR − 1.00 to 0.50) in *KIT* D816V-positive patients (*U* = 427.0, *p* = 0.843).

All patients with abnormal BMD underwent repeat DEXA scanning. The majority (*N* = 4/5) of patients with low BMD demonstrated spontaneous normalization of BMD on repeat DEXA scans. The mean interval between DEXA scans was 3 years. Only one (1.4%) patient with an associated eating disorder had persistent osteopenia.

Eleven (14.5%) patients from our DEXA scan cohort reported bone pain. We compared this to the total cohort of 229 patients, where this percentage was 11.3%. The median BMD *Z*-score of the TB among these individuals was − 0.4 (range − 2.1 to + 1.2) and BMD *Z*-score lumbar spine was − 0.9 (range − 1.2 to + 1.4). No correlation was found between bone pain and BMD.

Additionally, 5 patients experienced traumatic bone fractures, of which one pMPCM patient had a history of 2 fractures. However, all of them had normal BMD scores. We observed a moderate positive correlation between the reported number of symptoms and bone pain (Mann–Whitney *U* = 615.5, *p* < 0.001). To ensure that this association was not influenced by bone pain being included in the total symptom count, bone pain was filtered out of the symptom count. After this correction, the association remained significant (Mann–Whitney *U* = 579.0, *p* < 0.001).

There was no correlation between the number of symptoms and bone fractures (Mann–Whitney *U* = 174.0, *p* = 0.631). Spearman’s correlation analysis confirmed that there were no significant associations between bone fractures, number of symptoms, or tryptase levels (all *p* > 0.05). Additionally, no significant correlation was found between tryptase levels and bone pain (Mann–Whitney *U* = 141.0, *p* = 0.631).

## ISM patients

Patient characteristics are demonstrated in Table 3. BMD *Z*-scores are illustrated in Figs. 3 and 4.

All three ISM patients in our cohort had normal BMD scores. The BMD *Z*-scores for the TB were − 0.7, − 0.4, and + 2.4 (median − 0.4). The LS *Z*-scores were − 1.2, − 1.2, and + 0.1 (median − 1.2).

The median age at assessment was 8 years. Median tryptase levels in ISM patients were significantly higher than median tryptase levels in CM patients (Kruskal–Wallis test, *χ*^2^(4) = 24.25, *p* < 0.001). Pairwise comparisons using Bonferroni-adjusted Mann–Whitney *U* tests revealed that ISM patients had significantly higher tryptase levels compared to mastocytoma (*p* = 0.009), mMPCM (*p* = 0.002), and pMPCM (*p* < 0.001).

## Discussion

To our knowledge, this study is one of the first [20] to assess bone mineral density (BMD) in pediatric mastocytosis. We performed DEXA scans in 76 children with different forms of mastocytosis. The majority of these patients were diagnosed with CM, where MPCM was the most common subtype. ISM was rare in our study (*n* = 3). The distribution of different mastocytosis subtypes in this cohort is in line with previous pediatric series [1, 20]. 

Baseline serum tryptase levels varied significantly across subtypes, with the highest median levels found in mMPCM and the lowest in mastocytoma. The *KIT* D816V mutation was detected in a quarter of our patients, with the highest prevalence in mMPCM and ISM patients. Of note, in the absence of organomegaly, systematic bone marrow investigation was not uniformly performed in all patients with a positive peripheral blood *KIT* D816V result to exclude SM. Therefore, the presence of occult SM in some patients cannot be excluded.

In our cohort, 6.8% of CM patients had low BMD (osteopenia), representing a significant increase compared to the healthy reference population, where this is estimated at 2–3% [28]. However, none met the criteria for osteoporosis and median BMD *Z*-scores remained within normal limits [29].

No association was found between BMD and D816V mutation, tryptase levels, or other clinical characteristics, mirroring previous pediatric and adult cohorts [30, 31].

Osteopenia occurred only in MPCM, but this association was not significant. Contrary to our expectations, most cases occurred in pMPCM, despite mMPCM’s known association with SM and disease persistence in adulthood [8]. None of the ISM patients demonstrated osteopenia. However, the limited number of ISM and mMPCM patients limits our conclusions. Additionally, some patients currently classified as CM did not have a complete workup to rule out a current diagnosis of SM, representing potential bias.

Five children experienced traumatic fractures, all with normal BMD. Fractures were unrelated to symptom burden or tryptase levels. Skeletal fragility can contribute to trauma at all levels of impact [32]. However, differentiating traumatic fractures from fragility fractures is difficult in children, especially in unwitnessed trauma [33]. Since the trauma mechanism in our cohort is unknown, fragility fractures cannot be ruled out. However, with normal BMD in these patients, the latter is unlikely.

Interestingly, almost all osteopenic patients demonstrated spontaneous normalization of BMD on follow-up DEXA scans. Vitamin D levels were not systematically assessed at the time of DEXA scan. Since bone mass accrual continues through puberty, and children can experience catch-up bone mass acquisition, these transient changes could reflect normal bone maturation [28]. Longitudinal studies have demonstrated that bone mineral content and BMD increase substantially during puberty, with considerable inter-individual variation in timing of growth [34, 35]. Therefore, these transient reductions in BMD *Z*-scores may reflect physiologic variations in periods of rapid growth or delayed pubertal development rather than true osteopenia. These findings underscore the importance of longitudinal monitoring of BMD in children. Serial BMD measurements in children with mastocytosis could help to distinguish transient findings from true osteopenia [28].

Our data suggests that low BMD (osteopenia) is not a great concern in pediatric mastocytosis patients. However, isolated reports have described clinically relevant bone disease, including vertebral fractures, particularly in children with SM or clonal disease [20, 21].

These observations support a cautious approach to screening for bone involvement in pediatric mastocytosis. DEXA screening may be considered on an individual basis, with targeted screening reserved for those with systemic disease, extensive skin lesions persisting into adult age, or fractures suggestive of fragility.

In contrast to children, bone involvement is frequent in adult mastocytosis [19, 30, 36, 37]. In adult SM patients, osteoporosis (18–31%) and osteosclerosis (8–19%) are frequent, often leading to fractures, and the risk correlates with tryptase levels and disease severity [30, 31, 37, 38]. In adult CM, the data is limited, but osteoporosis is reported in 3.7%–15% of patients, while probably being associated with common osteoporosis risk factors [19, 30, 36, 37, 39]. Mastocytosis-related osteoporosis is characterized by focal osteolytic and osteosclerotic lesions, reduced trabeculae, and increased osteoid, osteoclast, and osteoblast numbers, indicating high bone turnover [19]. Bone turnover is influenced by histamine and various cytokines. Additionally, bone turnover parameters like bone-specific alkaline phosphatase (bALP) and C-terminal telopeptide (CTX) seem correlated with higher tryptase levels [19, 40].

Tryptase levels alone cannot explain the differences in BMD between adult and pediatric mastocytosis patients. In adult mastocytosis, median tryptase values range approximately from 10 to 30 ng/ml in CM and are substantially higher in adult SM, typically between 20 ng/ml and more than 200–500 ng/ml depending on the SM subtype [41, 42]. In children, these levels are generally lower. Median serum tryptase in mastocytoma and MPCM are usually below 20 ng/ml, while higher levels are reported In DCM (median levels approximately 67 mg/ml, IQR 24.9–154 ng/mL) [43]. In pediatric SM, median tryptase levels around 111 ng/ml (IQR 42–187 ng/mL) have been described [43]. Importantly, tryptase levels often decrease over time, in contrast to adult patients [13, 44]. The substantially higher tryptase levels in some types of adult SM may partly explain the greater risk of bone involvement compared with adult CM or pediatric mastocytosis [19, 31]. However, there is considerable overlap between adult and pediatric tryptase values, suggesting that tryptase alone cannot account for these differences. The observed differences in bone involvement may therefore relate to distinct disease biology rather than tryptase levels alone.

Our study was limited by the small number of patients with some mastocytosis subtypes, limiting conclusions. Given the relatively small number of children with DCM, mMPCM and SM (groups with higher tryptase levels and possibly higher risks), it might be too early to draw conclusions. Furthermore, we cannot exclude potential selection bias. Although DEXA scanning was not targeted toward patients with more severe cutaneous disease or elevated serum tryptase levels, inclusion was influenced by practical considerations, such as the child’s ability to undergo the procedure and alignment of follow-up appointments with our inclusion period. As a result, not all eligible patients underwent DEXA scanning. Nevertheless, the distribution of mastocytosis subtypes in our study cohort is in line with previously reported pediatric cohorts, suggesting that no major distortion toward a specific clinical phenotype occurred.

Determinants of pediatric BMD were not systematically assessed in this study, including vitamin D status, dietary calcium intake, body mass index, and level of physical activity. The absence of these potential confounders limits interpretation of our findings and precludes adjustment for non–disease-related influences on bone density. Additionally, the cross-sectional design prevents assessment of long-term outcomes. Longitudinal follow-up of BMD results was limited to patients with abnormal BMD results. Therefore, systematic follow-up for the entire cohort was not recorded.

Our data suggest that performing DEXA scans at a young age in children with CM may not be useful. Given the small number of patients with systemic disease, our findings should be interpreted with caution and do not allow firm conclusions regarding the optimal timing or indications for DEXA screening. Bone mineral density assessment may be considered on an individual basis, particularly in cases of suspected systemic involvement, when extensive lesions in CM persist into adult age, or when fractures suggestive of fragility are present.

Future research should focus on BMD in a larger, more representative pediatric mastocytosis cohort, preferably with longitudinal follow-up of bone density to further elucidate bone involvement in pediatric mastocytosis.

## Conclusion

Our findings suggest that bone involvement is not a great concern in children with cutaneous mastocytosis. Therefore, a DEXA scan could probably be reserved for children if systemic involvement is suspected, similar to adult patients [45–47]. Therefore, targeted screening could be useful in ISM or when extensive lesions in CM persist into adult age.

## Data Availability

The data used and analyzed in this study were extracted from patient medical records. These data are therefore not publicly available in order to protect patient confidentiality.

## Abbreviations

bALP: Bone-specific alkaline phosphatase
BMD: Bone mineral density
bsT: Baseline serum tryptase
CM: Cutaneous mastocytosis
c-kit: Tyrosine kinase receptor
CTX: C-terminal telopeptide
DCM: Diffuse cutaneous mastocytosis
DEXA: Dual-energy X-ray absorptiometry
ISM: Indolent systemic mastocytosis
IQR: Interquartile range
LS: Lumbar spine
mMPCM: Monomorphic maculopapular cutaneous mastocytosis
MPCM: Maculopapular cutaneous mastocytosis
pMPCM: Polymorphic maculopapular cutaneous mastocytosis
SD: Standard deviation
SM: Systemic mastocytosis
TB: Total body
VAF: Variant allelic frequency
